# THE LINK BETWEEN INTRAINDIVIDUAL VARIABILITY IN COGNITIVE PERFORMANCE AND MOBILITY IN CHRONIC STROKE

**DOI:** 10.1093/geroni/igae098.3053

**Published:** 2024-12-31

**Authors:** Vrinda Dimri, Nárlon Cássio Boa Sorte Silva, Guilherme Balbim, Teresa Liu-Ambrose

**Affiliations:** University of British Columbia, Vancouver, British Columbia, Canada; University of British Columbia, Vancouver, British Columbia, Canada; University of British Columbia, Vancouver, British Columbia, Canada; University of British Columbia, University of British Columbia, British Columbia, Canada

## Abstract

Intraindividual variability (IIV) is the within-person trial-to-trial variation in reaction time during cognitive tasks. Higher IIV represents reduced consistency in responses, which may be due to lapses in attention, and is associated with reduced mobility in older adults. IIV may also be a more sensitive measure of cognitive performance versus traditional summary scores. A stroke can significantly impact one’s cognitive function and mobility. Whether IIV in cognitive performance is associated with mobility among individuals who have experienced a stroke is unknown. We aimed to examine this relationship by using baseline data from a six-month single-blinded, 3-group parallel randomised controlled trial. Participants included community-dwelling adults (N= 119, 38.7% female) with a history of stroke, aged 55 years and older (mean= 70.71 years, SD= 8.59), able to walk 6 meters, and without dementia. Mobility was assessed based on timed up and go (TUG) performance. Residualised intra-individual standard deviation (rISD) was used as measure of IIV and computed using trial latencies of a computerised Stroop Task. TUG scores significantly predicted rISD of congruent (beta= 0.03, SE= 0.01, p-value = 0.001) and neutral trials (beta= 0.02, SE= 0.01, p-value = 0.04), but not incongruent trials (beta= 0.01, SE= 0.01, p-value = 0.18). However, these effects were not found when age was included as a covariate. Overall, these findings suggest that in adults with stroke, higher IIV measured from a simple reaction time task is associated with worse mobility. Further longitudinal studies are needed to determine if higher IIV is predictive of mobility decline post-stroke.

